# National cross-sectional survey on psychological impact on French nursing homes of the first lockdown during the COVID-19 pandemic as observed by psychologists, psychomotor, and occupational therapists

**DOI:** 10.3389/fpubh.2023.1290594

**Published:** 2023-12-27

**Authors:** Anne-Julie Vaillant-Ciszewicz, Bérengère Couturier, Lauriane Segaux, Florence Canouï-Poitrine, Olivier Guérin, Sylvie Bonin-Guillaume

**Affiliations:** ^1^Centre Hospitalier Universitaire de Nice, Nice, France; ^2^CoBTek EA7276, Nice, France; ^3^Université Paris Est Créteil, INSERM, IMRB, F-94010 Créteil, France; ^4^Centre Hospitalier Universitaire de Nice, Pôle Réhabilitation Autonomie et Vieillisssement, Université Côte d’Azur, Inserm U1081, CNR UMR 7284, Nice, France; ^5^Assistance Publique Hôpitaux de Marseille, Aix-Marseille Université, Marseille, France

**Keywords:** COVID-19 pandemic, psychological impact, lockdown, nursing homes, residents, health professionals, families

## Abstract

**Objectives:**

The main objective was to explore the psychological impact of the French lockdown during the first wave of the COVID-19 pandemic on nursing home residents, their relatives, and healthcare teams, as observed by mental health professionals.

**Design:**

A national online cross-sectional survey was conducted from May 11 to June 9, 2020.

**Setting and participants:**

Respondents were psychologists, psychomotor therapists, and occupational therapists (mental health professionals).

**Results:**

A total of 1,062 participants responded to the survey, encompassing 59.8% psychologists, 29.2% occupational therapists, and 11% psychomotor therapists. All mental health professionals felt fear (76.1%), fatigue and exhaustion (84.5%), and inability to manage the emotional burden (78.4%). In nursing homes with COVID-19 cases, residents felt significantly sadder (83.2%), more anxious (65.0%), experienced more anorexia (53.6%), resurgence of traumatic war memories (40.2%), and were more often disoriented (75.7%). The suffering of relatives did not vary between nursing homes with and without COVID-19 cases. The nursing staff was heavily impacted emotionally and was in need of psychological support particularly when working in nursing homes in a low COVID-19 spread zone with COVID-19 cases (41.8 vs. 34.6%).

**Conclusion and implications:**

Primary prevention must be implemented to limit the psychological consequences in the event of a new crisis and to prevent the risk of psychological decompensation of residents and teams in nursing homes.

## 1 Introduction

The emergence of COVID-19 on December 31, 2019 marked the inception of a global health crisis that rapidly escalated into a pandemic of unprecedented scale and impact. Aalto et al. (1) highlighted the profound influence of the virus within nursing homes (NHs), emphasizing its substantial impact on both mortality and morbidity.

Canoui-Poitrine et al. (2) observed a substantial surge in excess mortality in French NHs during the initial COVID-19 wave (March–May 2020). Within this period, French NHs reported a significant increase in fatalities, registering 13,505 additional deaths, reflecting a 43% rise in mortality rates. Overall estimations for the NHs population suggested they contributed to 51% of the excess deaths in the general population.

These findings underscore the considerable impact of the pandemic within these facilities and underscore the pressing need to comprehend its psychological repercussions on residents, families, and healthcare professionals. This challenging period was characterized by encounters with mortality, infections, resource scarcities, and strained healthcare services, all significantly impacting the mental well-being of the residents (3–5).

The initial COVID-19 lockdown in France, spanning from March 17th to May 11th, 2020, imposed stringent measures aimed at curtailing the spread of the virus. These measures encompassed restricted movement, closure of non-essential public spaces, and the transition to remote learning for educational institutions. This period significantly impacted NHs, where rigorous protocols were implemented to shield residents. These protocols included the suspension of family visits and the enforcement of strict health measures, exacerbating residents’ isolation and emotional distress. The lockdown revealed the specific challenges faced within NHs, highlighting residents’ vulnerability to the virus and intensifying emotional strain due to restricted social interactions.

Healthcare teams navigated complex conditions to ensure residents’ care while aimed to safeguard their health. Among the NHs staff, mental health professionals such as psychologists, psychomotor therapists, and occupational therapists played a pivotal role in prioritizing and enhancing residents’ psychological well-being, particularly during the lockdown period. To assess the emotional and psychological impact of the initial pandemic wave and lockdown on NH residents, families, and health professionals, we conducted a nationwide survey, gathering insights from mental health experts.

The initial wave of COVID-19 posed a lot of challenges to NHs, manifesting in high mortality rates and strained resources. Residents experienced mortality, infection, and limited medical support, significantly affecting their mental health. The absence of social connections further deteriorated well-being, particularly for those directly affected by COVID-19.

Additionally, the absence of social connections and activities further exacerbated the challenges faced by NH residents (6, 7), especially for those affected by COVID-19 (8). During the COVID-19 period, health restrictions and lockdown have impacted the whole world. Older people, particularly vulnerable, have been significantly affected by the crisis (9). Studies reported a high prevalence of psychological symptoms such as anxiety, depression, and fear during this period (10, 11). One of the primary causes of psychological distress among the older adult during the health crisis is attributed to social isolation (12, 13). According to Plagg et al. (14), the pandemic has triggered feelings of fear, loneliness, and social isolation among old people. These emotions could weaken their resilience; subsequently further compromising their psychological and subjective well-being.

Cerbara et al. (8) conducted a study shedding light on the interconnection between primary emotions and Maslow’s hierarchy of needs, accentuating the prioritization of essential physiological requisites during crisis scenarios. This association implies the prevalence of fear, anger, and sadness across diverse demographic segments, with anger and disgust particularly manifesting when individuals perceive a threat to meeting their fundamental needs, notably economic security. This investigation underscores the pivotal significance of comprehending emotional experiences within NHs settings amid the COVID-19 crisis, elucidating how altered fundamental needs have instigated a surge in adverse emotions within these facilities.

More recently, a study by Crespo-Martin et al. (15) highlighted the COVID-19 restrictions in NHs, which had a significant impact on residents. The authors found a disruption in residents’ routines leading to feelings of fear, loneliness, and a withdrawal from certain activities. However, the study also emphasized strong resources like social connections, spirituality, and gratitude. Another study conducted by Oliveira et al. (16) demonstrates that during the initial lockdown in Spain, the psychological well-being of NH residents was considered. The study indicates minimal psychological impact on residents, caregivers, and families due to significant resilience capacities (protective factors).

This study delves into the emotional impact of COVID-19 lockdowns in French NHs, focusing on healthcare professionals, residents, and families. The aim had been 2-fold: firstly, to comprehend the crisis-induced needs for refined crisis management strategies (17, 18) and secondly, to explore the psychological impact during unprecedented circumstances, shedding light on coping mechanisms (18). This research, conducted post-lockdown, provides crucial insights into vulnerable populations’ experiences (19, 20).

The study’s hypothesis focuses on the psychological challenges faced by residents, caregivers, and families, positing the emergence of emotional, behavioral, and cognitive symptoms due to the crisis’s exigencies (21). It captured real-time psychological states, distinct from declarative data. This field study gathered perceptions from mental health professionals, providing a unique insight into crisis-induced emotional impacts within NHs.

## 2 Methods

The authors used the Strengthening the Reporting of Observational Studies in Epidemiology (STROBE) cross-sectional reporting guidelines.

### 2.1 Study design and participants

This online and cross-sectional survey of mental health professionals (psychologists, occupational therapists, and psychomotor) working in nursing homes was conducted from May 11 to June 9, 2020, during the national lockdown in France. Its objective was to collect data on how the crisis was experienced from a psychological perspective by three key populations in NHs: residents, health professionals, and families.

#### 2.1.1 Tool creation

A questionnaire was developed to gather as much information as possible from professionals. This questionnaire was reviewed by a group of geriatricians and other professionals in the field as nurses, psychologists, and occupational therapists. The questionnaire was anonymized and made available online, and a call for participation was launched on a national scale. Prior to the questionnaire development, a sample of 50 NHs in the South of France was gathered. As part of establishing COVID-19 telephone hotlines in geriatrics, psychological aspects concerning residents, healthcare workers, and families were used to create a list of symptoms for study. This list then formed the basis for crafting the survey.

#### 2.1.2 Inclusion criteria

Eligibility criteria for this study required all respondents to be professionals actively engaged in NHs settings during crisis periods in France. Participants must speak, write, and understand the French language. They must also have internet access to respond to the online questionnaire.

#### 2.1.3 Exclusion criteria

Those individuals not actively employed in nursing homes during crisis periods or declining to provide consent were excluded from participating in the survey. Individuals who do not have proficiency in the language (expression and comprehension in French) and those who do not have access to the internet are not included.

#### 2.1.4 Recruitments of participants

Participants were recruited anonymously through a solicitation for participation distributed across diverse channels, including professional societies and associations associated with French nursing homes. Prior to participation, all respondents provided explicit consent by digitally confirming their willingness to engage in the survey. Their commitment extended to completing the comprehensive questionnaire addressing their perceptions during crisis situations.

A convenient sampling method was employed to recruit participants. The team conducted a follow-up to enhance response rates for the study. Efforts were made to bolster response rates by leveraging an extensive professional network and engaging with professional societies in the field.

### 2.2 Setting

The study focused on NH residents, families, and healthcare teams (nursing staff and mental health professionals) during the first wave of COVID-19 in France and overseas departments. French NHs provide accommodation, medico-social services (such as meals and laundry), and medical, nursing, and psychosocial care to dependent residents who require regular medical and nurse attention (3).

About 7,400 NHs were listed in France (2022). These establishments, designed to accommodate older adult with reduced autonomy, are distributed among private, public, and associative entities. This diversified distribution between private, public, and associative management contributes to varying operational modes and healthcare practices within these establishments, shaping the experiences of residents and healthcare staff. These facilities have varying capacities ranging from a few dozen to several hundred beds, reflecting the diverse needs and accommodation capacities for dependent older adult in France. The distribution is not homogeneous across the national territory.

Characteristics of the NHs were extracted from the respondents’ answers, but due to the anonymization of the collected data, it was not possible to identify the NHs where respondents worked.

### 2.3 Outcomes

The outcomes were the psychological conditions (e.g., symptoms of anxiety and depression) of nursing home residents, families, and healthcare professionals (nursing staff and mental health professionals).

The variables examined for residents encompass heightened levels of anxiety, increased sadness, fear of viral infection, concerns regarding contamination, negative ideation, thoughts of self-harm, withdrawal tendencies, decreased appetite, behavioral disturbances related to productivity (specifically observed in people with neurocognitive disorders), temporal and spatial disorientation, recollection of traumatic events such as wars, and separation anxiety from caregivers.

For families, we measured the following variables using the same method as for the residents. The questions had focused on understanding the implemented health measures in NHs, the fear of infecting loved ones, satisfaction with the communication means in place, the expression of significant emotional distress due to the lack of contact with relatives, and an expressed need for more psychological support.

For the healthcare teams, measurements had been taken of several variables such as the presence of high emotional disturbance, increased stress and anxiety, more depression, a greater work overload, an emotional burden at work, fear of being infected by the virus and fear of contaminating the residents, and the need for more psychological support.

Regarding the respondents (mental health professionals), various questions had been asked, such as those related to fear during that crisis period, sleep disturbances, sadness, discouragement, fatigue and burnout, and feeling less effective at work.

Authors had taken into account several potential biases in the study, for instance biases related to the subjectivity of responses concerning the study’s objectives. All method-related biases (inherent to the chosen questionnaire methodology) as well as the strengths of the study were discussed in the discussion section. All the NHs that had volunteered to participate in our survey were able to take part in the questionnaires.

### 2.4 Data collection

An online data collection tool using Google Forms was developed by multidisciplinary experts involved in the COVID-19 committee managed by the French Geriatric and Gerontology Society (SFGG) during the pandemic. The survey was tested, reviewed, and validated by the SFGG’s academic board. A link to the Google Forms survey was widely disseminated to all SFGG members, to members of national professional organizations (psychologists, occupational therapists, and nursing home physicians), and to the academic institutions training occupational and psychomotor therapists. Each questionnaire was filled out online anonymously by participants. Submission of a completed survey was considered as agreement to participate. A reminder was sent the week before closing the survey.

The questionnaire consisted of five sections. The first section included nine questions on the respondents’ activities within the nursing home (profession, working time, and work in specific Alzheimer units) and characteristics of the nursing home (number of residents, type of nursing home, location, and COVID-19 status of residents).

The next three sections each included five statements using a five-point Likert scale (ranging from 1: “not at all” to 5: “absolutely”) and explored the psychological impact of the pandemic and the lockdown on different sub-populations of respondents: nursing home residents (13 questions), relatives (six questions), and nursing staff (nine questions). Finally, respondents were asked about their own perceptions (six questions with the following response modalities: “yes”/“no”/“no opinion”; Supplementary Table).

### 2.5 Data sources

The data from the Google Forms were anonymous and automatically stored in a spreadsheet on a Google Drive then analyzed after completion of the study. Only researchers in charge of the analysis had access to the data. The data were securely stored in Google Forms in an anonymous manner.

### 2.6 Ethics and regulatory framework

The survey was approved by the Nice University Hospital Geriatric and Alzheimer Clinical Ethics Committee (June 8, 2020). All personal data of the participants has been deleted to ensure the anonymity of the data. In the context of the study, a brief paragraph was provided to inform participants about the study. The text explicitly states that data are collected anonymously. Additionally, the data storage for a duration of 15 years, in compliance with French regulations, is also mentioned. Participants agreed to the ethical rules by clicking the “Next” button, which granted them access to the questionnaire. If a participant declined, they could not complete the questionnaire. Participants did not receive compensation for their involvement in the study. Ensuring the rights of individuals taking part in the study was a particular priority.

### 2.7 Statistical methods

Each analyzed variable corresponded to the answer to one question from the survey questionnaire. Continuous variables were described as medians [interquartile ranges (IQR)]. Categorical variables were described as numbers (percentages). Questions with five statements using a five-point Likert scale were analyzed as a binary variable by grouping answer modalities (1–3 and 4–5 on the Likert scale), a positive response (i.e., major impact) corresponding to answers ranging from 4 to 5, in view of the non-homogeneous distribution of answers in the different statements for each analyzed variable. NHs were assigned to a geographical area. The Statistics department of the French health ministry (DREES, 2021) (22) has mapped every NH with at least one resident affected by COVID-19 during the first lockdown. A high COVID-19 spread zone was defined as 50% or more NH with at least one COVID-19 resident. Based on this map and the location of respondents’ NH, a new variable, spread zone (high versus low) was created. Responders were divided into two groups based on the COVID-19 status of the NH residents (cluster or not, cluster being defined by the presence of at least one infected resident) and location of the NH in a spread zone (high vs. low). Variables for conducting subgroup analysis were selected on the basis of both statistical (significant differences *p* < 0.05 in univariate analyses with respondents’ characteristics) and epidemiological considerations. The groups’ characteristics were compared using Pearson’s chi-squared test or Fisher’s exact test (as appropriate) for categorical variables, and the Kruskal-Wallis test for continuous variables. Multivariable logistic regression models were used to systematically adjust for organizational variables (structure of the nursing home, number of residents, and amount of time the mental health professionals work in the nursing home), as potential confounding factors. All tests were two-tailed, and the threshold for statistical significance was set at *p* < 0.05. Missing data were taken into account as follows: incomplete questionnaires concerning variables used for subgroup analysis were excluded. Also, for questions with the following response modalities: “yes”/“no”/“no opinion” (*n* = 6), respondents not expressing an opinion, the response “no opinion” was used and they were not included in the analyses. All statistical computations were performed using Stata software (version 16.1).

## 3 Results

### 3.1 Sample characteristics

Among the 1,084 professionals who filled out the questionnaire, COVID-19 spread zone (geographic location of the NH; missing data, *n* = 4) or COVID-19 status (missing data, *n* = 18) were not available for 22 respondents, which led to an analyzable population of 1,062. Data from these 22 respondents were excluded from the analyses.

**Figure 1:**
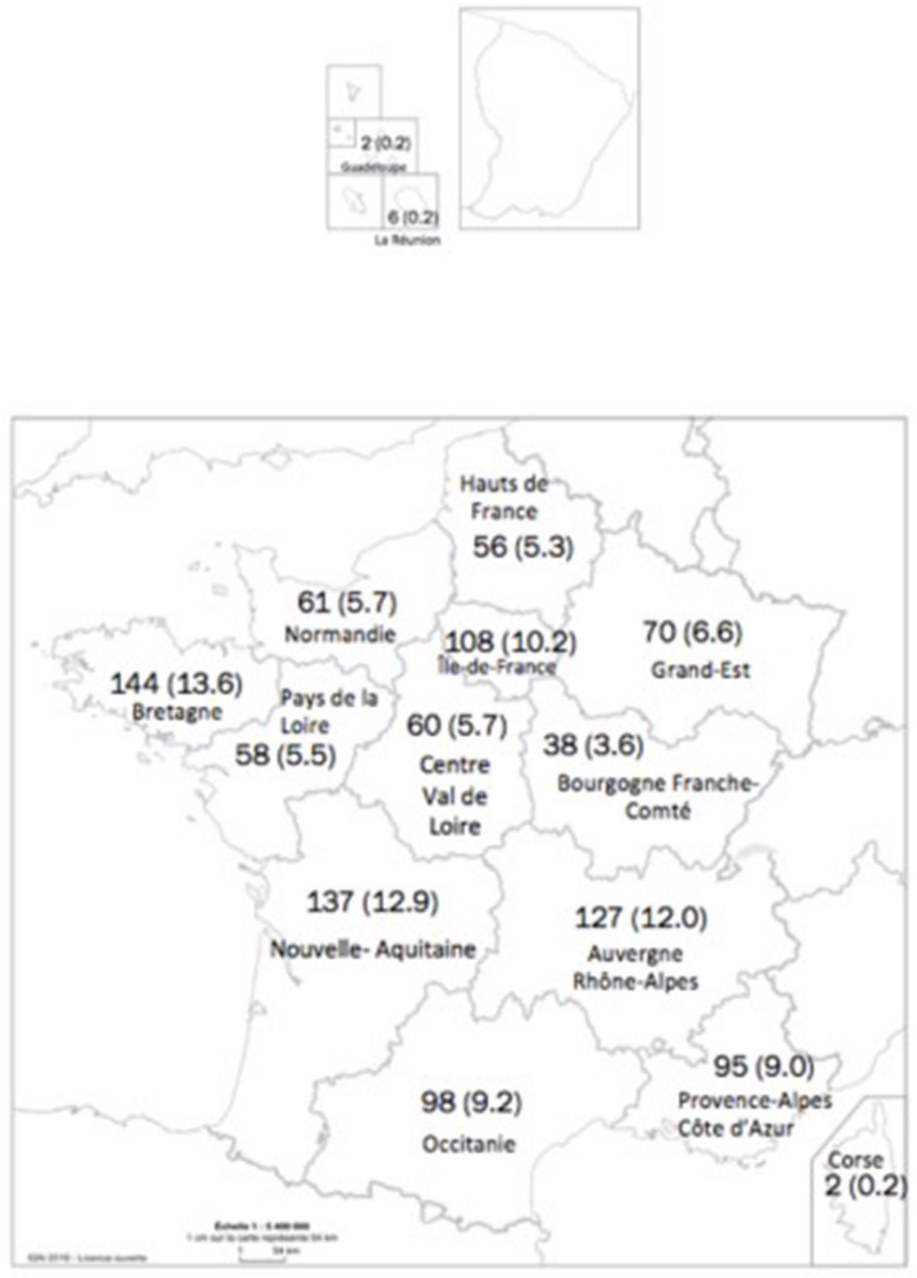
French regions represented in the survey and number of respondents.

Of these, 59.8% were psychologists, 29.2% occupational therapists, and 11.0% psychomotor therapists (five respondents did not complete this item). Most of them (52.0%) worked in a public nursing home; and 56.2% worked in specific Alzheimer units. The overall median number of residents living in a private nursing home was 81 (Q1–Q3:70–95), and 94 (Q1–Q3: 75–150) in a public NH. The proportion of time the respondents were working in the NH was 10–20% for 8.5%; 30–50% for 38.4%; and > 50% for 53.1%. All French regions were represented (mainland and French overseas departments; Figure 1). One third of the respondents (*n* = 372) had worked while COVID-19 residents were present in the NH; and in 34.0% (*n* = 316) of the cases, the nursing home was located in a high-spread zone (a large part of the north and east of France).

### 3.2 Lockdown and pandemic consequences on the mental health of nursing home residents

88.1% of residents suffered from isolation and not seeing their relatives, 80.1% felt greater sadness, and 70.9% were spatially and temporally disoriented. Residents living in a NH where COVID-19 cases had occurred (compared to those in nursing homes without COVID-19 cases), were more likely to fear being contaminated by the virus (23.5 vs. 16.1%, *p* adjusted = 0.001), to develop anorexia (53.6 vs. 40.2%, *p* adjusted <0.001), sadness (83.2 vs. 78.4%, *p* adjusted = 0.026), anxiety (65.0 vs. 61.0%, *p* adjusted = 0.027), resurgent memories (40.2 vs. 35.0%, *p* adjusted = 0.041), or be disoriented because of the lockdown (75.7 vs. 68.2%, *p* adjusted = 0.003; Table 1).

**Table 1:** Lockdown and pandemic consequences on the mental health of nursing home (NH) residents, residents’ relatives, nursing staff, and respondents (nursing home mental health professionals).

	Total population	NH without COVID-19 residents	NH with COVID-19 residents	*p* value^*^	Adjusted value of p^**^
	*N* = 1,062	*N* = 690	*N* = 372		
Concerning residents^***^					
Greater anxiety *n* (%) (*N* = 1,060)	661 (62.4)	420 (61.0)	241 (65.0)	0.20	**0.027**
Greater sadness *n* (%) (*N* = 1,059)	848 (80.1)	540 (78.4)	308 (83.2)	0.059	**0.026**
Fear of COVID-19 infection for themselves *n* (%) (*N* = 1,059)	198 (18.7)	111 (16.1)	87 (23.5)	**0.004**	**0.001**
Fear of contaminating their close relatives (*N* = 1,060)	431 (40.7)	269 (39.0)	162 (43.7)	0.14	**0.007**
More pessimistic or suicidal ideation *n* (%) (*N* = 1,058)	515 (48.7)	335 (48.7)	180 (48.7)	0.99	0.62
More renunciation behaviors *n* (%) (*N* = 1,055)	606 (57.4)	384 (55.9)	222 (60.3)	0.17	0.13
More anorexia symptoms, *n* (%) (N = 1,057)	475 (44.9)	276 (40.2)	199 (53.6)	**<0.001**	**<0.001**
More productive behavioral symptoms in residents with neurocognitive disorders, *n* (%) (*N* = 1,055)	552 (52.3)	350 (51.0)	202 (54.9)	0.22	0.07
More disorientation in time and space, *n* (%) (*N* = 1,057)	749 (70.9)	468 (68.2)	281 (75.7)	**0.010**	**0.003**
More traumatic memories of the second world war (*N* = 1,057)	389 (36.8)	240 (35.0)	149 (40.2)	0.10	**0.041**
Significant suffering from the separation from their close relatives (*N* = 1,060)	934 (88.1)	608 (88.1)	326 (88.1)	1	0.96
Concerning relatives^***^					
Understand the health measures (*N* = 1,061)	723 (68.1)	474 (68.8)	249 (66.9)	0.54	**0.041**
Fear of contaminating their relative (*N* = 1,060)	340 (32.1)	224 (32.6)	116 (31.2)	0.65	0.95
Satisfied with the technical devices developed by the nursing home to communicate with their relative, *n* (%) (*N* = 1,057)	832 (78.7)	556 (81.1)	276 (74.4)	**0.012**	**0.004**
Significant emotional suffering, *n* (%) (*N* = 1,059)	888 (83.9)	568 (82.4)	320 (86.5)	0.09	0.10
Expressed the need for more psychological support, *n* (%) (*N* = 1,051)	588 (56.0)	386 (56.6)	202 (54.7)	0.56	**0.032**
Concerning nursing staff^***^					
Higher emotional distress, *n* (%) (*N* = 1,060)	622 (58.7)	375 (54.5)	247 (66.4)	**<0.001**	**<0.001**
More stressed and anxious, *n* (%) (*N* = 1,060)	806 (76.0)	494 (71.8)	312 (83.9)	**<0.001**	**<0.001**
More depressed, *n* (%) (*N* = 1,060)	377 (35.6)	218 (31.7)	159 (42.7)	**<0.001**	**<0.001**
Greater work overload, *n* (%) (*N* = 1,061)	697 (65.7)	429 (62.3)	268 (72.0)	**0.001**	**0.001**
Emotional burden at work, *n* (%) (*N* = 1,061)	738 (69.6)	451 (65.5)	287 (77.2)	**<0.001**	**<0.001**
Fear of being contaminated, *n* (%) (*N* = 1,060)	608 (57.4)	358 (51.9)	250 (67.6)	**<0.001**	**<0.001**
Fear of contaminating their residents, *n* (%) (*N* = 1,059)	791 (74.7)	522 (75.8)	269 (72.7)	0.28	0.34
Need for psychological support, *n* (%) (*N* = 1,056)	382 (36.2)	232 (33.9)	150 (40.3)	**0.039**	0.62
Concerning themselves (mental health professionals)					
Fear, *n* (%) (*N* = 1,040)	791 (76.1)	506 (74.9)	285 (78.3)	0.21	0.13
Disturbed sleep *n* (%) (*N* = 1,022)	560 (54.8)	338 (51.2)	222 (61.3)	**0.002**	**0.002**
Greater sadness, *n* (%) (*N* = 1,032)	351 (34.0)	200 (29.8)	151 (41.9)	**<0.001**	**<0.001**
Discouragement, *n* (%) (*N* = 1,033)	367 (35.5)	218 (32.3)	149 (41.5)	**0.003**	**0.003**
Fatigue and/or burnout, *n* (%) (*N* = 1,048)	885 (84.5)	552 (81.4)	333 (90.0)	**<0.001**	**0.002**
Loss of efficiency at work, *n* (%) (*N* = 1,001)	483 (48.3)	290 (44.5)	193 (55.3)	**0.001**	**0.035**

^*^CHI-2 test. ^**^Univariate analysis with adjustment for the variable structure of the nursing home, number of residents, and percentage of time spent by the professionals in the nursing home. ^***^Corresponds to respondents who answered 4 or 5 on a Likert scale.

In the subgroup analysis (Table 2), some consequences appeared more frequently for residents living in NHs with COVID-19 cases in high-spread zones, notably anxiety (66.5 vs. 55.0%, *p* adjusted = 0.005), fear of being contaminated (25.7 vs. 10.9%, *p* adjusted < 0.001), resurgence of Second World War memories (41.3 vs. 30.2%, *p* adjusted = 0.018), and greater temporal disorientation (77.0 vs. 67.2%, *p* adjusted < 0.001).

**Table 2:** Main results according to the location of the respondents’ nursing home and COVID-19 status of residents.

	Total population in low-spread zone^*^	Total population in high-spread zone^*^	*p* value^**^	Adjusted value of p^***^	Population in low-spread zone		*p* value	Adjusted *p* value	Population in high-spread zone		*p* value	Adjusted *p* value
	*N* = 701	*N* = 361			*N* = 701				*N* = 361			
					NH without COVID-19 residents	NH with COVID-19 residents			NH without COVID-19 residents	NH with COVID-19 residents		
					*N* = 560	*N* = 141			*N* = 130	*N* = 231		
Concerning residents^****^												
Greater anxiety, *n* (%) (*N* = 1,060/*N* = 701/*N* = 359)	437 (62.3)	224 (62.4)	0.99	0.93	349 (62.3)	88 (62.4)	0.98	0.85	71 (55.0)	153 (66.5)	**0.031**	**0.005**
Greater sadness, *n* (%) (*N* = 1,059/*N* = 701/*N* = 358)	555 (79.2)	293 (82.8)	0.30	0. 33	438 (78.2)	117 (83.0)	0.21	0.07	102 (79.1)	191 (83.4)	0.31	0.46
Fear of COVID-19 infection for themselves, *n* (%) (*N* = 1,059/*N* = 700/*N* = 359)	125 (17.9)	73 (20.3)	0.33	0.20	97 (17.4)	28 (19.9)	0.49	0. 13	14 (10.9)	59 (25.7)	**0.001**	**<0.001**
Fear of contaminating their close relatives (*N* = 1,060/*N* = 701/*N* = 359)	282 (40.2)	149 (41.5)	0.69	0.70	219 (39.1)	63 (44.7)	0.23	0.14	50 (38.8)	99 (43.0)	0.43	0.07
More pessimistic or suicidal ideation, *n* (%) (*N* = 1,058/*N* = 700/*N* = 358)	330 (47.1)	185 (51.7)	0.16	0.12	266 (47.6)	64 (45.4)	0.64	0.07	69 (53.5)	116 (50.7)	0.61	0.18
More renunciation behaviors, *n* (%) (*N* = 1,055/*N* = 698/*N* = 357)	400 (57.3)	206 (57.7)	0.90	0.88	315 (56.5)	85 (60.7)	0.36	0.22	69 (53.5)	137 (60.1)	0.23	0.45
More anorexia symptoms, *n* (%) (*N* = 1,057/*N* = 699/*N* = 358)	294 (42.1)	181 (50.6)	**0.009**	**0.012**	221 (39.6)	73 (51.8)	0.009	**0.015**	55 (43.0)	126 (54.8)	**0.032**	0.28
More productive behavioral symptoms in residents with neurocognitive disorders, *n* (%) (*N* = 1,055/*N* = 698/*N* = 357)	354 (50.7)	198 (55.5)	0.14	0.16	279 (50.0)	75 (53.6)	0.45	0.22	71 (55.0)	127 (55.7)	0.90	0.39
More disorientation in time and space, *n* (%) (*N* = 1,057/*N* = 699/*N* = 358)	486 (69.5)	263 (73.5)	0.18	0.19	382 (68.5)	104 (73.8)	0.22	0.08	86 (67.2)	177 (77.0)	**0.045**	**<0.001**
More frequent traumatic memories of the second world war (*N* = 1,057/*N* = 698/*N* = 359)	255 (36.5)	134 (37.3)	0.80	0.19	201 (36.1)	54 (38.3)	0.63	0.27	39 (30.2)	95 (41.3)	**0.037**	**0.018**
Significant suffering from the separation from their close relatives (*N* = 1,060/*N* = 701/*N* = 359)	625 (89.2)	309 (86.1)	0.14	0.18	495 (88.4)	130 (92.2)	0.19	0.28	113 (86.9)	196 (85.6)	0.73	0.97
Concerning relatives^****^												
Relatives understand the health measures (*N* = 1,061/*N* = 701/*N* = 360)	484 (69.0)	239 (66.4)	0.38	0.46	385 (68.8)	99 (70.2)	0.74	0.59	89 (69.0)	150 (64.9)	0.44	0.40
Fear of contaminating their relative (*N* = 1,060/*N* = 700/*N* = 360)	227 (32.4)	113 (31.4)	0.73	0.86	187 (33.5)	40 (28.4)	0.25	0.53	37 (28.7)	76 (32.9)	0.41	0.23
Satisfaction with technical devices developed by the nursing home to communicate with their relative *n* (%) (*N* = 1,057/*N* = 699/*N* = 358)	570 (81.6)	262 (73.2)	**0.002**	**0.002**	457 (81.9)	113 (81.0)	0.63	0.56	99 (77.3)	163 (70.9)	0.19	**0.048**
Significant emotional suffering, *n* (%) (*N* = 1,059/*N* = 701/*N* = 358)	581 (82.9)	307 (85.8)	0.23	0.19	462 (82.5)	119 (84.4)	0.59	0. 74	106 (82.2)	201 (87.8)	0.15	0.98
Expressed the need for more psychological support, *n* (%) (*N* = 1,051/*N* = 695/*N* = 356)	392 (56.4)	196 (55.1)	0.68	0. 23	316 (56.9)	76 (54.3)	0.57	0. 63	70 (55.1)	126 (55.0)	0.99	1
Concerning nursing staff^****^												
Greater emotional distress, *n* (%) (*N* = 1,060/*N* = 699/*N* = 361)	389 (55.7)	233 (64.5)	**0.005**	0. 81	306 (54.8)	83 (58.9)	0.39	0.17	69 (53.1)	164 (71.0)	**0.001**	**0.006**
More stressed and anxious, *n* (%) (*N* = 1,060/*N* = 699/*N* = 361)	510 (73.0)	296 (82.0)	**0.001**	**0.001**	397 (71.2)	113 (80.1)	**0.032**	**0. 031**	97 (74.6)	199 (86.2)	**0.006**	**0.005**
More depressed, *n* (%) (*N* = 1,060/*N* = 699/*N* = 361)	228 (32.6)	149 (41.3)	**0.005**	**0.002**	179 (32.1)	49 (34.8)	0.55	0. 58	39 (30.0)	110 (47.6)	**0.001**	**0.001**
Greater work overload, *n* (%) (*N* = 1,061/*N* = 700/*N* = 361)	447 (63.9)	250 (69.3)	0.08	0.06	343 (61.4)	104 (73.8)	**0.006**	**0.008**	86 (66.2)	164 (71.0)	0.34	0.26
Report an emotional burden at work, *n* (%) (*N* = 1,061/*N* = 700/*N* = 361)	469 (67.0)	269 (74.5)	**0.012**	**0.013**	360 (64.4)	109 (77.3)	**0.004**	0.16	91 (70.0)	178 (77.1)	0.14	0.23
Fear of being contaminated, *n* (%) (*N* = 1,060/*N* = 700/*N* = 360)	381 (54.4)	227 (63.1)	**0.007**	**0.009**	292 (52.1)	89 (63.6)	**0.015**	**0.006**	66 (50.8)	161 (70.0)	**<0.001**	**0.001**
Fear of contaminating their residents, *n* (%) (*N* = 1,059/*N* = 699/*N* = 360)	527 (75.4)	264 (73.3)	0.47	0. 47	422 (75.5)	105 (75.0)	0.90	1	100 (76.9)	164 (71.3)	0.25	0.37
Need for psychological support, *n* (%) (*N* = 1,056/*N* = 696/*N* = 360)	251 (36.1)	131 (36.4)	0.92	0. 12	192 (34.6)	59 (41.8)	0.11	**0.048**	40 (31.0)	91 (39.4)	0.11	0. 54
Concerning mental health professionals												
Fear, *n* (%) (*N* = 1,040/*N* = 689/*N* = 351)	507 (73.6)	284 (80.9)	**0.009**	**0.011**	407 (74.1)	100 (71.4)	0.52	0.40	99 (78.0)	185 (82.6)	0.29	0. 52
Disturbed sleep, *n* (%) (*N* = 1,022/*N* = 672/*N* = 350)	357 (53.1)	203 (58.0)	0.14	0.18	271 (50.5)	86 (63.7)	**0.006**	**0. 001**	67 (54.5)	136 (59.9)	0.33	0.63
Greater sadness, *n* (%) (*N* = 1,032/*N* = 683/*N* = 349)	199 (29.1)	152 (43.6)	**<0.001**	**<0.001**	155 (28.4)	44 (32.1)	0.39	0.24	45 (35.7)	107 (48.0)	**0.026**	**0.029**
Discouragement, *n* (%) (*N* = 1,033/*N* = 681/*N* = 352)	212 (31.1)	155 (44.0)	**<0.001**	**<0.001**	167 (30.6)	45 (33.3)	0.54	0.41	51 (39.8)	104 (46.4)	0.23	0.25
Greater fatigue and burnout, *n* (%) (*N* = 1,048/*N* = 692/*N* = 356)	571 (82.5)	314 (88.2)	**0.016**	**0. 021**	446 (80.9)	125 (88.7)	**0.032**	0. 23	106 (83.5)	208 (90.8)	**0.039**	**0.029**
Loss of efficiency at work, *n* (%) (*N* = 1,001/*N* = 655/*N* = 346)	290 (44.3)	193 (55.8)	**<0.001**	**0. 002**	229 (43.5)	61 (47.7)	0.39	0.44	61 (48.8)	132 (59.7)	**0.049**	0.22

^*^Classification of the location of respondents’ practice based on the DREES map “Proportion of nursing homes affected in wave 1” (20). ^**^Chi-2 test. ^***^Univariate analysis with adjustment for the variable structure of the nursing home, number of residents and percentage of time spent by respondents in the nursing home. ^****^Respondents who answered 4 or 5 on the Likert scale. Bold values correspond to *p* < 0.05.

### 3.3 Pandemic consequences on the mental health and needs of the residents’ relatives

83.9% of residents’ relatives emotionally suffered (sadness and stress) because of the residents’ lockdown. Furthermore, 56.0% of relatives and loved ones frequently expressed the need for more psychological support. In adjusted analyses, the suffering of relatives did not vary between respondents in nursing homes with COVID-19 and without COVID-19 cases among residents (86.5 vs. 82.4%, *p* adjusted = 0.10; Table 1). There was also no difference in the subgroup analysis (Table 2).

### 3.4 Pandemic consequences on the mental health of nursing home health professionals

76.0% of NH health professionals felt stressed and anxious because of the pandemic and 74.7% feared contaminating their residents. Working in a nursing home with COVID-19 cases significantly increased their emotional suffering. They reported being stressed and anxious (83.9 vs. 71.8%, *p* adjusted < 0.001), feeling depressed (42.7 vs. 31.7%, *p* adjusted < 0.001), emotionally burdened (77.2 vs. 65.5%, *p* adjusted < 0.001), experiencing emotional suffering (66.4 vs. 54.5%, *p* adjusted < 0.001), fear of being contaminated (67.6 vs. 51.9%, *p* adjusted < 0.001), and work overload (72.0 vs. 62.3%, *p* adjusted = 0.001; Table 1).

More than one third of the nursing staff (36.2%) was in need of psychological support, especially those working in a nursing home with COVID-19 cases, in a low-spread zone (41.8 vs. 34.6%, *p* adjusted = 0.048; Table 2).

The nursing staff working in nursing homes located in a high-spread zone were more affected overall than those working in nursing homes located in a low-spread zone, particularly when there were COVID-19 cases in the NH, except for fear of contaminating the residents (71.3 vs. 76.9%, *p* adjusted = 0.37), which was high in all cases. In the subgroup analysis, the nursing staff working in a nursing home located in a high-spread zone felt more frequently depressed (47.6 vs. 30.0%, *p* adjusted = 0.001) and experienced greater emotional distress when there were COVID-19 cases (71.0 vs. 53.1%, *p* adjusted = 0.006), whereas their work load felt heavier when working in a nursing home with COVID-19 cases in a low-spread zone (73.8 vs. 61.4%, *p* adjusted = 0.008; Table 2).

### 3.5 Pandemic consequences on the mental health of respondents

Respondents were also asked to report their personal feelings during the pandemic. 76.1% of mental health professionals experienced fear and 84.5% reported fatigue and exhaustion. Mental health professionals working in NHs with COVID-19 cases felt sadder (41.9 vs. 29.8, *p* adjusted < 0.001), more discouraged (41.5 vs. 32.3%, *p* adjusted = 0.003), more exhausted (90.0 vs. 81.4%, *p* adjusted = 0.002), and complained about disturbed sleep (61.3 vs. 51.2%, *p* adjusted = 0.002) and loss of efficiency at work (55.3 vs. 44.5%, *p* adjusted = 0.035; Table 1).

Respondents working in high-spread areas were overall more affected compared to those working in a low-spread area except for sleep complaints (58.0 vs. 53.1%, *p* adjusted = 0.18; Table 2). Overall, 21.7% of them considered that they were able to identify and manage the emotional burden of being healthcare professionals.

## 4 Discussion

This research indeed delved into the emotional and psychological ramifications of the first COVID-19 lockdown (23) on mental health professionals, residents, caregivers, and families within French NHs (24). It had taken into account variations between NHs with documented COVID-19 cases and those situated in areas characterized by high vs. low infection spread during France’s initial COVID-19 surge. Employing real-time questionnaire-based methodologies, the study captured crucial insights into the psychological well-being and functioning of these diverse populations amid the lockdown’s challenges and uncertainties.

This research, by segmenting the NHs based on COVID-19 incidence and transmission rates, aimed to decipher the distinct impacts of the pandemic and lockdown on various strata within the NH community. The utilization of real-time data collection methods, likely questionnaire-based, provided a nuanced understanding of the emotional and psychological dynamics experienced by mental health professionals, residents, caregivers, and families during that critical period of pandemic-induced lockdowns.

This investigation demonstrated robustness in evaluating the psychological implications during the initial COVID-19 surge, emphasizing data completeness and confounding factor adjustments. The examination of influencing elements and expert scrutiny of survey items enhanced the study’s reliability. However, limitations encompassed biases inherent in online surveys and the absence of detailed individual profiles. Given the crisis context, mitigating non-response biases associated with online questionnaires had been challenging. The study, while innovative, lacked qualitative data integration in item construction, which could have bolstered its depth. The absence of psychometric tools was due to the urgency for a concise field questionnaire, impacting methodological aspects for quick responses (25).

Despite limitations, the study offered real-time insights during a challenging period, although reproducing results might have posed difficulties. The study’s strength lay in its broad representation across the entirety of France, reflected in a substantial response rate (1,660 responses) from the 7,400 NHs in France in 2022. It was also important to note that the questionnaire items had been constructed based on feedback from a small number of healthcare professionals (psychologists, occupational therapists, and psychomotor therapists) at that time. This was a strength because even though the questionnaire was not based on scientific literature (which was very limited or non-existent at that time), it originated from professionals working directly in the field.

Our findings indicate that emotional impacts were associated with virus exposure within NHs and their geographic locations. NHs in high spread areas with COVID-19 cases reported more adverse effects on residents, including fear, exhaustion, and depressive symptoms, consistent with previous research. Mental health professionals expressed emotional strain, seeking psychological support, particularly in NHs managing COVID-19 cases. Here is what our results were able to highlight among the various studied populations. We compared our results with the scientific literature. This step allowed us to describe the psychological impact of the pandemic and lockdown on residents, families, loved ones, as well as healthcare professionals.

- Residents exhibited increased behavioral disturbances due to halted visitations, emphasizing the importance of understanding the needs of those with cognitive impairments or mood disorders during crises. Concerning NHs residents, a German literature review utilized the PRISMA method to comprehend the psychosocial impact of the global pandemic and its confinement on residents (11). The findings are compelling, as out of 756 studies, the authors selected 15. Residents primarily experienced loneliness, grief, and depression linked to worldwide health restrictions. These observations, even if we did not specifically study the grief variable, align with our own findings.
- Caregivers faced overwhelming situations and lacked necessary tools to support residents, colleagues, and families, hinting at a need for comprehensive crisis management training. An article by Zhao et al. (26) demonstrated, as our survey also did, that healthcare professionals have suffered from the situation of confinement and Covid-19. In this study, 147 healthcare professionals were surveyed, with 21.8% reporting feelings of depression and 24.5% experiencing anxiety. In our study, anxiety scores were higher (*n* = 1,062), reaching 76% in a larger sample size compared to the cited study. Regarding depressive states, the scores were at 42.7%. These results highlight the significant impact of the pandemic situation and its confinement on healthcare staff in France and globally. The studies also demonstrate the importance of employing coping strategies to better manage the health crisis and its psychological impact. In our study, 36.2% of respondents expressed a need for psychological support, aligning with Zhao et al.’s research (26), which highlights the significance of positive coping strategies and social support for healthcare teams.
- While families expressed contentment with communication channels (digital meetings for instance), reassessing communication modes during crises may mitigate psychological consequences. Despite geographic variations, families experienced distress but utilized digital communication tools to maintain connections with the older adult. Some Dutch scientific research (27) has indicated that relatives of residents were satisfied with communication methods when facilitated by a nurse-initiated telephone call or through visits behind glass or at a distance outdoors. Our study also revealed that most relatives appeared satisfied with the array of communication methods implemented during that period in the nursing home. Respondents (*n* = 1997) in this study (27) experienced feelings of loneliness and a sense of missing their loved ones, reported at 76%, which aligns with our findings. Similarly, in the Dutch study, relatives also expressed feelings of sadness at a rate of 66%, which closely corresponds to our results (83.2% of sadness and stress).

Research conducted by Hugelius et al. (28) and Bezinger et al. (11) corroborate our study’s outcomes, emphasizing the profound implications of COVID-related constraints on the mental health of residents (manifesting as depression and loneliness) and the well-being of families (characterized by ethical dilemmas regarding visitations and fear of contamination). These studies underscore the necessity of integrating emotional responses into strategies aimed at preventing pandemics. Surprisingly, the study by Crespo-Martin et al. (15) shows that the psychological impact of the first lockdown in Spain, assessed at three different times (beginning, middle, and end), appears to be minor compared to the findings of our study. Therefore, it would be interesting to understand why Spanish NHs exhibit greater resilience to the crisis than those in France.

The lockdown’s negative effects were observed across NHs, affecting residents, families, caregivers, and mental health professionals. Finally, the international literature seems to support the same conclusions as our article within the studied populations (9–11, 26–29). Tailored regional support aligned with NH characteristics is imperative, including training mental health professionals in coping strategies and psychosocial interventions (27). Raising awareness among psychologists to identify mental risks can benefit residents and NH teams. The results confirm significant psychological distress within the analyzed populations, consistent with existing literature, enabling proposed psycho-behavioral management strategies during crises.

Suggestions for targeted interventions may involve offering psychological support techniques to professionals, establishing a supportive culture, regular emotional monitoring, and involving psychologists trained in evidence-based therapies, such as Cognitive and Behavioral Therapies (CBT). These interventions could encompass ongoing training programs for healthcare staff, or older adult (29), aiming to develop specific skills in emotional management and psychological support to address the unique challenges encountered in care facilities. Implementing protocols to foster an organizational culture that encourages the expression of emotions and peer support could also be a promising intervention approach.

Additionally, instituting systems for regular emotional monitoring would enable early detection of emotional needs and difficulties, facilitating prompt and targeted intervention such as social support (16). Finally, integrating psychologists specialized in evidence-based therapies like CBT could enhance available resources to provide adequate and tailored psychological support to residents, healthcare staff, and families in care facilities. These intervention suggestions are grounded in a holistic approach aiming to address multiple and complex emotional needs encountered in a healthcare setting during a crisis.

## 5 Conclusion

Our study highlights the emotional burden and psychological impact of the first COVID-19 lockdown on French NHs. Although recommendations have since been published to better optimize NH organization in the event of a new health crisis for both residents and relatives, there is still a lot to be done to protect nurses and mental health professionals from the psychological impact by providing professional support during and after the crisis. Going forward, future research should aim for more regular surveys among healthcare professionals, residents, and families to better identify psychological triggers during crises (continuous assessment). It is also crucial to provide mental health professionals with training in cognitive-behavioral strategies and emotional regulation for improved crisis response. Lastly, establishing support and listening systems for professionals appears to be necessary.

## Data availability statement

The raw data supporting the conclusions of this article will be made available by the authors, without undue reservation.

## Ethics statement

The studies involving humans were approved by Chu de nice: comité éthique azuréen. The studies were conducted in accordance with the local legislation and institutional requirements. Written informed consent for participation was not required from the participants or the participants’ legal guardians/next of kin because it was an online survey. Written informed consent was not obtained from the individual(s) for the publication of any potentially identifiable images or data included in this article because it was an online survey about psychological facts.

## Author contributions

A-JV-C: Review, Writing – original draft, Writing – review & editing. BC: Methodology, Review, Writing – original draft. LS: Data curation, Methodology, Writing – review & editing. FC-P: Data curation, Methodology, Supervision, Validation, Visualization, Writing – review & editing. OG: Conceptualization, Funding acquisition, Review, Supervision, Writing – review & editing. SB-G: Conceptualization, Funding acquisition, Project administration, Resources, Review, Supervision, Validation, Writing – original draft.
