# Supplementary material for: National cross-sectional survey on psychological impact on French nursing homes of the first lockdown during the COVID-19 pandemic as observed by psychologists, psychomotor, and occupational therapists
Source: Front Public Health. 2023 Dec 27;11:1290594. doi: 10.3389/fpubh.2023.1290594 (PMC10778814; doi:10.3389/fpubh.2023.1290594)
Supplement: Supplementary file 1 [file Data_Sheet_1.PDF]

Questionnaire IPCE : Impact Psychologique du Covid-19 en EHPAD

Sondage auprès des psychologues, ergothérapeutes et psychomotriciens en EHPAD concernant l' impact psychologique du confinement et de la crise Covid-19.

Présentation du questionnaire et des consignes importantes :

Mesdames, Messieurs,
Vous êtes psychologue, ergothérapeute ou psychomotricien en EHPAD. La société française de Gériatrie et Gérontologie (SFGG) vous sollicite afin de répondre à une enquête concernant l'impact psychologique du Covid-19 dans votre établissement.
Les réponses au questionnaire sont anonymes, elles permettrons de générer des données sur l'impact psychologique de cette crise sanitaire en EHPAD.
Votre participation à ce questionnaire doit prendre une dizaine de minutes maximum.
Nous allons vous proposer des affirmations et des questions concernant les résidents, les familles et les soignants.

ATTENTION, SI VOUS EXERCEZ DANS PLUSIEURS ÉTABLISSEMENT, IL EST IMPORTANT DE REMPLIR UN QUESTIONNAIRE PAR ÉTABLISSEMENT.

Nous vous remercions chaleureusement pour votre participation.

Pour la SFGG, le Conseil Scientifique.

Informations générales

Merci de bien vouloir remplir la section ci-dessous.

1. Exercez-vous actuellement en EHPAD?

   Une seule réponse possible.

   - Oui
   - Non

2. Quel poste occupez-vous en EHPAD?

   Une seule réponse possible.

   - psychologue
   - ergothérapeute
   - psychomotricien

3. Temps d'activité au sein de l'EHPAD :

Une seule réponse possible.

- 10 à 20 %
- 30 à 50%
- Plus de 50 %

4. Dans quel département se trouve l'EHPAD dans lequel vous exercez?

5. Code postal :

6. Nombre de résidents dans l'EHPAD :

7. Votre EHPAD est :

Une seule réponse possible.

- une structure publique
- une structure privée
- autre

8. Avez-vous eu des résidents Covid positif?

Une seule réponse possible.

- Oui
- Non
- Ne se prononce pas

9. Travaillez-vous en unité protégée?

Une seule réponse possible.

- ( ) Oui
- ( ) Non
- ( ) Ne se prononce pas

Les résidents et l'impact psychologique de la crise sanitaire et du confinement en EHPAD

Merci de répondre aux affirmations suivantes par une échelle de 1 à 5.

10. Depuis le début du confinement, les résidents vous paraissent plus inquiets.

Une seule réponse possible.

	1	2	3	4	5	
Pas du tout	( )	( )	( )	( )	( )	Tout à fait

11. Depuis le début du confinement, les résidents vous paraissent plus tristes.

Une seule réponse possible.

	1	2	3	4	5	
Pas du tout	( )	( )	( )	( )	( )	Tout à fait

12. Les résidents évoquent souvent la peur de l'infection à Covid-19 pour eux-mêmes.

Une seule réponse possible.

	1	2	3	4	5	
Pas du tout	( )	( )	( )	( )	( )	Tout à fait

13. Les résidents vous font part de leur crainte que leurs proches soient infectés au Covid-19.

Une seule réponse possible.

	1	2	3	4	5	
Pas du tout	◯	◯	◯	◯	◯	Tout à fait

14. Les résidents ont plus d'idées noires ou d'idées suicidaires depuis le début du confinement.

Une seule réponse possible.

	1	2	3	4	5	
Pas du tout	◯	◯	◯	◯	◯	Tout à fait

15. Les résidents ont plus de comportement de renoncement (soins et activités usuelles) depuis le début du confinement.

Une seule réponse possible.

	1	2	3	4	5	
Pas du tout	◯	◯	◯	◯	◯	Tout à fait

16. Les résidents présentent davantage de signes d'anorexie depuis le début du confinement.

Une seule réponse possible.

	1	2	3	4	5	
Pas du tout	◯	◯	◯	◯	◯	Tout à fait

17. Les résidents qui ont des troubles neurocognitifs manifestent davantage de troubles du comportement perturbateurs (agitation, agressivité) depuis l'instauration du confinement.

Une seule réponse possible.

	1	2	3	4	5	
Pas du tout	◯	◯	◯	◯	◯	Tout à fait

18. Le confinement engendre une aggravation de la désorientation dans le temps et dans l'espace pour les résidents.

Une seule réponse possible.

	1	2	3	4	5	
Pas du tout	◯	◯	◯	◯	◯	Tout à fait

19. Depuis le début du confinement, les résidents évoquent plus fréquemment les souvenirs en lien avec la guerre.

Une seule réponse possible.

	1	2	3	4	5	
Pas du tout	◯	◯	◯	◯	◯	Tout à fait

20. Les résidents montrent une souffrance significative vis à vis de l'absence ou de l'éloignement de leurs proches.

Une seule réponse possible.

	1	2	3	4	5	
Pas du tout	◯	◯	◯	◯	◯	Tout à fait

21. D'après vous, la mise en place des visites en EHPAD avec mesures barrières est possible pour les résidents avec troubles cognitifs.

Une seule réponse possible.

	1	2	3	4	5	
Pas du tout	◯	◯	◯	◯	◯	Tout à fait

22. Pour les résidents réinstaurer les visites des familles améliore leur bien-être psychologique.

Une seule réponse possible.

	1	2	3	4	5	
Pas du tout	◯	◯	◯	◯	◯	Tout à fait

Les familles et l'impact psychologique de la crise sanitaire en EHPAD

Merci de répondre aux affirmations suivantes par une échelle de 1 à 5.

23. De manière générale les familles/les proches comprennent les mesures mises en place.

Une seule réponse possible.

	1	2	3	4	5	
Pas du tout	◯	◯	◯	◯	◯	Tout à fait

24. Les familles/proches craignent de contaminer leurs proches lors des visites.

Une seule réponse possible.

	1	2	3	4	5	
Pas du tout	◯	◯	◯	◯	◯	Tout à fait

25. Les familles/les proches sont satisfaits des moyens de communication mis en place par l'EHPAD (skype, zoom etc).

Une seule réponse possible.

	1	2	3	4	5	
Pas du tout	◯	◯	◯	◯	◯	Tout à fait

26. Les familles/les proches expriment une souffrance émotionnelle significative (inquiétudes, tristesse) en lien avec le confinement du résident.

Une seule réponse possible.

	1	2	3	4	5	
Pas du tout	◯	◯	◯	◯	◯	Tout à fait

27. Actuellement, les familles/les proches expriment un besoin plus important de soutien psychologique.

Une seule réponse possible.

	1	2	3	4	5	
Pas du tout	◯	◯	◯	◯	◯	Tout à fait

28. La mise en place des visites en EHPAD est bénéfique sur le plan émotionnel pour les familles/proches.

Une seule réponse possible.

	1	2	3	4	5	
Pas du tout	◯	◯	◯	◯	◯	Tout à fait

Les équipes soignantes et l'impact psychologique de la crise sanitaire en EHPAD.

Merci de répondre aux affirmations suivantes par une échelle de 1 à 5.

29. Les équipes soignantes manifestent plus de troubles émotionnels depuis le début de l'épidémie.

Une seule réponse possible.

	1	2	3	4	5	
Pas du tout						Tout à fait

30. Les membres des équipes sont davantage stressés et anxieux depuis le début de la crise.

Une seule réponse possible.

	1	2	3	4	5	
Pas du tout						Tout à fait

31. Les membres des équipes sont davantage déprimés depuis le début de la crise.

Une seule réponse possible.

	1	2	3	4	5	
Pas du tout						Tout à fait

32. Actuellement, les membres de l'équipe décrivent une surcharge de travail.

Une seule réponse possible.

	1	2	3	4	5	
Pas du tout						Tout à fait

33. Actuellement, les membres de l'équipe décrivent un épuisement.

Une seule réponse possible.

	1	2	3	4	5	
Pas du tout	◯	◯	◯	◯	◯	Tout à fait

34. Les équipes expriment une peur pour elles-même vis à vis de la contamination.

Une seule réponse possible.

	1	2	3	4	5	
Pas du tout	◯	◯	◯	◯	◯	Tout à fait

35. Les équipes craignent de contaminer les résidents.

Une seule réponse possible.

	1	2	3	4	5	
Pas du tout	◯	◯	◯	◯	◯	Tout à fait

36. Les équipes sont en demande de soutien psychologique.

Une seule réponse possible.

	1	2	3	4	5	
Pas du tout	◯	◯	◯	◯	◯	Tout à fait

37. Vous estimez avoir les moyens pour identifier les troubles émotionnels chez les soignants et leur proposer une prise en charge adaptée.

Une seule réponse possible.

	1	2	3	4	5	
Pas du tout	⬭	⬭	⬭	⬭	⬭	Tout à fait

A propos de vous et de vos ressentis lors de la crise sanitaire en EHPAD.

Merci de bien vouloir répondre aux questions suivantes vous concernant.

38. Avez-vous ressenti de la peur depuis le début de la crise?

Une seule réponse possible.

- Oui
- Non
- Ne se prononce pas

39. Dormez-vous aussi bien que d'habitude depuis le début de la crise?

Une seule réponse possible.

- Oui
- Non
- Ne se prononce pas

40. Etes vous plus triste depuis le début de la crise?

Une seule réponse possible.

- Oui
- Non
- Ne se prononce pas

41. Vous sentez-vous découragé depuis le début de la crise?

Une seule réponse possible.

- Oui
- Non
- Ne se prononce pas

42. Vous sentez vous davantage fatigué ou épuisé depuis le début de la crise?

Une seule réponse possible.

- Oui
- Non
- Ne se prononce pas

43. Avez-vous l'impression d'être aussi efficace que d'habitude au travail?

Une seule réponse possible.

- Oui
- Non
- Ne se prononce pas

Félicitations et merci, vous avez presque terminé

Merci d'avoir pris le temps de répondre à ce sondage, encore un dernier point :

44. Je souhaite recevoir les résultats du sondage par e-mail

Plusieurs réponses possibles.

- [ ] Oui : je note mon mail dans la case "autre"
- [ ] Non merci, je ne souhaite pas recevoir les résultats du sondage.

Autre : [ ] ______________________
